# Assessment of an Intra-Articular Drain in Arthroscopy-Assisted Anterior Cruciate Ligament Reconstruction

**DOI:** 10.7759/cureus.16928

**Published:** 2021-08-05

**Authors:** Sharib Shamim, Sudhir S Kushwaha, Kumar Shantanu, Garima Maurya, Iram Arshad

**Affiliations:** 1 Orthopedic Surgery, Era's Lucknow Medical College and Hospital, Lucknow, IND; 2 Orthopedics, All India Institute of Medical Sciences, Gorakhpur, IND; 3 Orthopedics, King George's Medical University, Lucknow, IND; 4 Obstetrics and Gynecology, Baba Raghav Das (BRD) Medical College, Gorakhpur, IND; 5 Pathology, Core Diagnostics, Lucknow, IND

**Keywords:** arthroscopy, anterior cruciate ligament, drain

## Abstract

Introduction

The use of a drain after various types of arthroscopic surgeries has long been debated. Whether a drain offers an advantage in terms of pain, swelling, and functional outcome after arthroscopy-assisted reconstruction of the anterior cruciate ligament (ACL) needs to be investigated. This study was designed to assess the validity of the use of an intra-articular drain after routine arthroscopic ACL reconstruction and to assess the various complications associated with its use.

Material and methods

Forty-four patients (group I included patients for whom an intra-articular drain was used and group II included patients for whom an intra-articular drain was not used) diagnosed with ACL injury were included in the study. The patients in group I had a drain placed inside the joint, while those in group II had a drain placed outside the joint cavity but the drain placement was such that there remained no patient or observer bias.

Results

Outcome assessment was performed on days one, two, and three followed by weeks one, four, and eight, and six months after surgery by determining a visual analog pain (VAS) score. The assessment was also done for the range of motion (ROM) in terms of loss of flexion and extension with a hand-held goniometer, knee hemarthrosis, and thigh circumference. Although there was a difference in both the groups in terms of the above-mentioned parameters in the early post-operative period, the difference becomes insignificant at the final follow-up at six months.

Conclusion

From this study, we conclude that putting an intra-articular drain after ACL reconstruction offers no advantage in terms of functional outcome in the long term.

## Introduction

The knee joint is the most commonly injured of all joints and an anterior cruciate ligament (ACL) tear is the most common serious ligamentous injury to the knee joint [1]. Arthroscopy-assisted reconstruction of a torn ACL has now become the gold standard in the treatment for this condition. Various authors have concluded that post-operative hemarthrosis accounts for the majority of complications associated with arthroscopy-assisted surgery and certain studies have found that it accounts for up to 24% of complications in various arthroscopic procedures [2]. Locally acting measures to reduce post-operative hemarthrosis mainly include compression dressings. A significant proportion of arthroscopic surgeons currently also use intra-articular drains after various arthroscopic procedures. The usual justification for the use of a drain is reducing patient pain, hemarthrosis, and associated complications [3].

However, the use of a drain remains a topic of debate. Surgeons are divided on the use of intra-articular drains following arthroscopic surgery. Surgeons who are in favor of using a drain advocate that the use of an intra-articular drain decreases the risk of hemarthrosis, adhesions, and joint stiffness. On the other hand, surgeons who are against using a drain routinely, after arthroscopic ACL reconstruction, advocate that using a drain might increase the risk of infection or can cause damage to the articular cartilage/graft during the insertion of the drain [4]. Therefore, this study aimed to assess the validity of the use of an intra-articular drain after routine arthroscopic ACL reconstruction and to assess the various complications associated with its use.

## Materials and methods

This study was conducted in the Department of Orthopedics, Era’s Lucknow Medical College, Lucknow. Forty-four patients (22 in each group) with ACL injuries who attended the orthopedic outpatient department (OPD) between March 2017 and March 2018 were included in the study. Necessary permissions were taken from specific ethical and scientific committees (this study is approved by Era's Lucknow Medical College Institutional Human Ethics Committee {IHEC} - #ELMC/EC/R_cell/2017/185). All ethical issues were considered before including participants in this study. In a previous study by El Khalifa et al., it was found that the mean flexion loss in the first week of follow-up in the drain group was 18.7 ± 0.48, while that in the non-drain group was 19.2 ± 0.69 [5]. Using these values as a reference, the minimum required sample size, with 80% power and a 5% level of significance, was 22 patients in each group.

Pre-operative

The study population was divided into two groups, and a drain was inserted for all the patients. The patients in one group had a post-operative intra-articular drain, while those in the other group had a drain at the donor site to avoid patient-related biasing. Groups were randomized by a simple random table. A detailed history was obtained and clinical examination was conducted by anterior drawer, Lachman, and pivot shift test for all patients. In addition, X-ray and MR imaging were performed for all participants to confirm the diagnosis.

All skeletally mature patients who had an ACL tear, confirmed by the Lachman test and MRI, with or without concomitant meniscal injury were included in the study. Written informed consent was obtained from all the patients who participated in the study. Patients with multi-ligament injury, revision surgery, and bleeding disorders were excluded from the study. After the ACL reconstructions, a post-operative drain was placed in all the patients, and the drain was placed in such a manner that there would be no observer bias among the patients of the two groups. A drain was placed inside the joint space in one group of patients and at the donor site in another group and there was no communication with the joint cavity. However, the skin exit site was close to the graft harvesting site in both groups. In the intra-articular drain group, a part of the tube was placed into a subcutaneous tunnel from the graft harvesting site up to the anteromedial portal to enter the joint space through the portal; for those in the donor site drain group, the drain tube ended at the donor site itself. The drain was taken out from a site close to the graft harvesting incision site in all cases. The drain was removed after two days and the drain exit site was similar in both the groups in due course of time, thereby avoiding observer bias. Post-operative joint pain, hemarthrosis, and range of movements were compared between the two groups. Clinical parameters like pain were evaluated using the visual analog pain (VAS) and hemarthrosis was measured using the clinical grading of hemarthrosis by Coupens and Yates (Table 1) [2]. We also measured the difference in thigh girth and range of motion (ROM), including loss of extension and flexion.

**Table 1:** Clinical grading of hemarthrosis. Source: Coupens and Yates [2].

Clinical grading of hemarthrosis	
Grade 0	No detectable fluid
Grade 1	Fluid present with wave
Grade 2	Fluid palpable in supra-patellar pouch
Grade 3	Ballotable patella
Grade 4	Tense hemarthrosis

Surgical technique

All the patients in the study were treated by the same team of surgeons and the arthroscopic “anatomical” single-bundle ACL reconstruction technique was used in all the patients. A single intravenous dose of antibiotic was given to all the patients before the surgery. A pneumatic tourniquet was used in all the patients and exsanguination was done before inflation set in. The ACL was reconstructed using a quadruple hamstring graft, which was harvested from the ipsilateral knee. Tightrope RT (Naples, FL: Arthrex) was used for fixation of the graft on the femoral side and bio-absorbable screws on the tibial site.

Post-operative care and follow-up

Post-operatively, IV antibiotics were given for three days in all the cases. A long knee brace was provided for patients in both groups. Knee mobilization exercises were begun on day three after the removal of the drain. Priority was given to the recovery of full extension. A continued passive motion was also used to help the patients with flexion. Further, active static quadriceps exercises were initiated as soon as the patient recovered from anesthesia. Partial weight-bearing with crutches/walkers was allowed for the next one month. Walking aids were maintained as necessary, till full quadricep control was re-established. Thereafter, standard ACL reconstruction rehabilitation protocol was followed. The patients were told that they could engage in sports activities only after six months. Follow-up was done on days one, two, and three followed by weeks one, four, and eight, and then six months after surgery. No follow-up loss was reported during this period.

Statistical methods

The statistical analysis in this study was done using SPSS version 16 (Chicago, IL: SPSS Inc.). Quantitative variables were presented as mean ± SD and comparison was done using the unpaired t-test/Mann-Whitney U test and paired t-test/Wilcoxon tests. Qualitative variables were compared using chi-square/Fisher’s exact test. A p-value of <0.05 was considered significant.

## Results

After following the inclusion and exclusion criteria, we enrolled 44 patients in this study. These patients were divided into two groups of 22 each - group I included patients with an intra-articular drain and group II included patients without an intra-articular drain.

The mean age (years) of the patients in group I was 29.68 ± 8.20, while in group II was 29.23 ± 7.70, and, therefore, the cohort was comparable for age (p = 0.851). There was eccentric gender distribution in both groups, with the number of males (n = 40) being much more than the number of females (n = 4), and this can be attributed to less frequent outdoor activity and lower participation in sports among females.

The mean duration of time from injury to the surgery was comparable in both the groups (group I: 4.91 ± 2.43; group II: 4.18 ± 2.22) because of the random allocation of the patients (p < 0.330). The duration of hospital stays and tourniquet time was comparable in both groups (Table 2). The average drain amount in group I was 127.68 ± 44.03 ml, while it was 32.95 ± 18.94 ml in group II, and the difference was significant (p < 0.001). The reason for this can be attributed to the direct connection of the drain with the intra-articular cavity in group I, while the collection in group II was because of the collection from the muscle and fat planes.

**Table 2:** Patients characteristics.

Characteristics	Group I	Group II	p-Value
Number of patients	22	22	-
Mean age (years)	29.68 ± 8.20	29.23 ± 7.70	0.851
Sex (male)	21	19	-
Sex (female)	1	3	-
Time from injury to surgery (months)	4.91 ± 2.43	4.18 ± 2.22	0.330
Hospital stay (days)	8.14 ± 4.24	7.73 ± 3.74	0.664
Tourniquet time (minutes)	50.23 ± 7.27	47.18 ± 4.23	0.100
Cutaneous anesthesia (number)	3	1	-
Drain amount (ml)	127.68 ±44.03	32.95 ± 18.94	<0.001

Hemarthrosis

On day two, the drain was removed for all the patients in both groups. On day one, the mean grade of hemarthrosis was 2.55 ± 0.74, while on day two it was 2.73 ± 0.63 for patients of group II; the patients in group I had no assessable hemarthrosis. After removal of the drain on day two, we measured the hemarthrosis on day three, and the mean grade of hemarthrosis was found to be significantly lower in group I (p = 0.005). The statistically significant difference lasted till the first week post-operative.

During the fourth week follow-up, we noticed that hemarthrosis was found only in 14 patients and the mean amount of hemarthrosis in patients of both groups was equal (seven in each group). After the fourth-week follow-up, grade 1 hemarthrosis was found in two patients of group II (Table 3). Further, we noticed tense hemarthrosis in two patients from group II and their knees were aspirated on post-operative day three before they were sent home.

**Table 3:** Mean grade of hemarthrosis in groups I and II.

Hemarthrosis	Group I		Group II		p-Value
	n	Mean ± SD	n	Mean ± SD	
Day 1	0	-	22	2.55 ± 0.74	-
Day2	0	-	21	2.73 ± 0.63	-
Day3	22	1.82 ± 0.73	22	2.50 ± 0.80	0.005
1st week	22	1.33 ± 0.48	22	1.68 ± 0.57	0.036
4th week	7	1.00 ± 0.00	7	1.00 ± 0.00	1.00
8th week	0	-	2	1.00 ± 0.00	-
6 months	0	-	0	-	-

Thigh circumference

**Figure 1:**
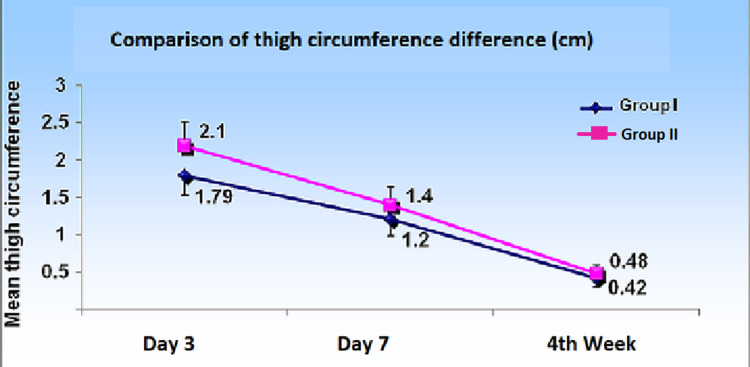
Thigh circumference difference (centimeters) among the groups over the follow-up period till fourth week.

The difference in the pre-operative and post-operative thigh circumference was measured on days three, seven, and on the week four follow-up, with the intention to account for the presence of hemarthrosis in the knee joint, which would change limb girth. We measured the supra-patellar thigh girth at 5 cm above the upper pole of the patella. On day three, the mean difference from pre-operative value was 1.79 ± 0.26 in group I patients while it was 2.19 ± 0.32 in group II patients, and the difference was statistically significant (p < 0.001). The difference was significant on follow-up on day seven as well. On the week four follow-up, this value became statistically insignificant (Figure 1).

Post-operative pain

**Figure 2:**
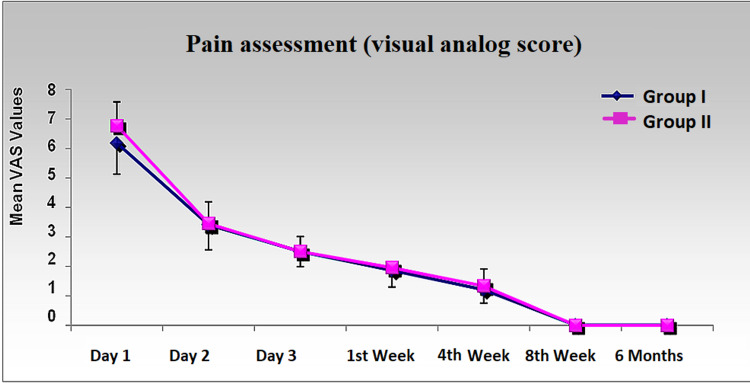
Difference in post-operative pain measured by visual analog score from day one to six months.

Post-operative pain was measured by the VAS in our study on days one, two, and three, consecutively, followed by weeks one, four, eight, and at six months (Figure 2). We found statistically significant pain differences in the two groups on day one itself. The pain was comparatively better tolerated in group I patients on day one, with a mean of 6.18 ± 1.05 in comparison to the patients who did not have a drain in the joint cavity (6.77 ± 0.81). On day two, patients in both groups had no statistically significant pain difference. Both groups showed a significant decrease in pain over time (days one to seven).

Range of motion

In our study, we measured the range of motion by loss of extension and loss of flexion. Measurements were taken with a handheld dual-arm goniometer. 

Loss of Flexion

**Figure 3:**
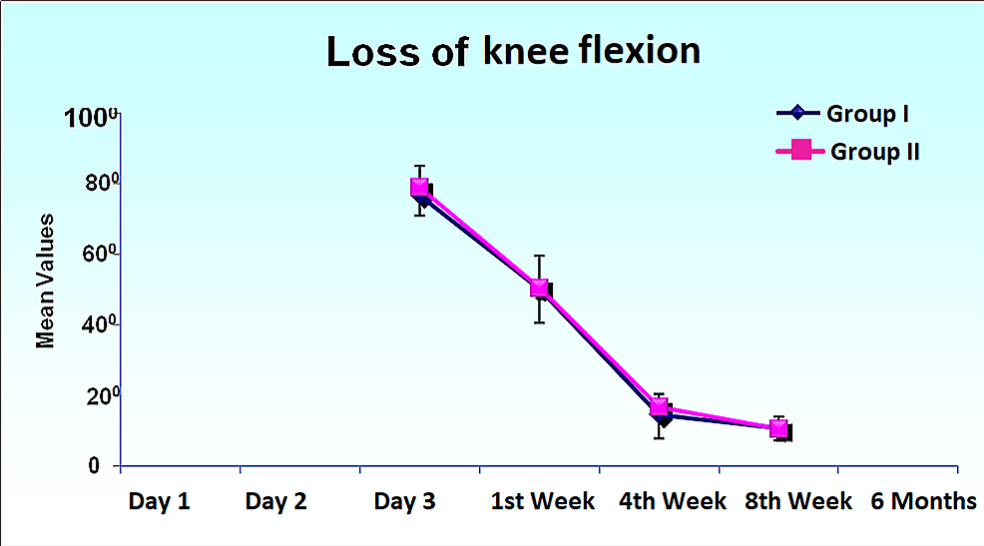
Comparison of loss of knee flexion in degrees over a period of day one to six months’ post-operative.

The loss of flexion was measured from day three, weeks one, four, and eight, and at six months. On day three, the mean loss of flexion was 76.55 ± 5.60 in group I patients, while it was 78.77 ± 6.23 in group II patients, and it remained statistically insignificant throughout the follow-up period (Figure 3).

Loss of Extension

**Figure 4:**
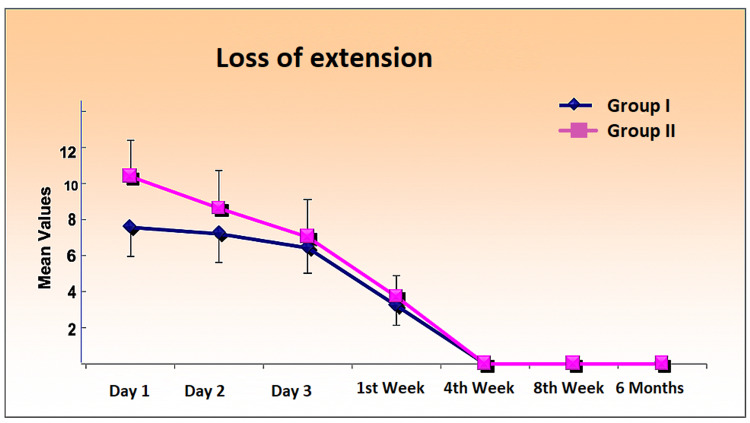
Comparison of loss of knee extension in degrees over a period day one to six months post-operative.

Knee extension was initiated just after the effect of anesthesia subsided. On the first post-operative day, mean loss of extension was 7.59 ± 1.65 for patients in group I, while it was 10.41 ± 2.02 in patients in group II; it was statistically significant on day two as well. This significant difference disappeared from day three onward, with full extension in all the patients from the first month onward (Figure 4).

## Discussion

The methods to reduce post-operative swelling and hemarthrosis, a common complication following an arthroscopic procedure, include compression dressings and tourniquets [6]. Despite the use of these dressings, tourniquets, and drains, hemarthrosis after an arthroscopic procedure continues to be the most commonly reported complication. Hemarthrosis has been associated with increased scar formation, decreased range of motion, and subsequent synovitis in patients who have undergone various arthroscopic procedures. The formation of hematoma can also lead to an increased risk of tissue compression, which can result in wound infection or neurologic compromise [7].

Currently, there is a mounting controversy regarding the use of a post-operative intra-articular drain after arthroscopic ACL reconstruction. There are numerous theoretical advantages to the use of prophylactic drains in orthopedic procedures. Serous fluids, which can accumulate in closed wound beds, are deficient in opsonic proteins. These proteins are necessary for binding to antigens and inducing phagocytosis by macrophages and neutrophils. Hematoma is also believed to be a good bacterial culture medium and its accumulation inside a wound provides an opportunity for the development of infection. The iron products in a wound hematoma and, more specifically, a hemarthrosis can adversely affect both chondrocytes and matrix.

Closed-suction drainage, like the Hemovac drainage system used in this study, involves a soft silicone elastomeric negative pressure system to remove fluids. The closed aspect of the system theoretically decreases the likelihood of external contamination. The placement of prophylactic drains may eliminate contamination and bacterial proliferation in surgical wounds. However, there could be increased bleeding in drained wounds. Drain dysfunction or non-function provides little or no evacuation of fluid from the wound but continues to cause many of the risks of drain use. This possibility must be evaluated while considering prophylactic drain usage.

McCormack et al. conducted a study on 118 patients with arthroscopic ACL reconstruction and measured hemarthrosis on the first, fourth, and eighth weeks after surgery and found no significant difference between the two groups in terms of the pain and range of outcome [8]. El Khalifa et al. included forty consecutive arthroscopic ACL reconstruction patients, and they were randomized alternately for either intra-articular suction drain group or non-drain group [5]. They concluded that the grade of hemarthrosis was lower in patients in the drain group than in those in the non-drain group. Further, 17 patients out of the 20 in the non-drain group were aspirated for hemarthrosis in the first two days, while only three patients in the drain group required aspiration on the first day. None of the patients of either group developed hemarthrosis in due course of time throughout the follow-up period.

Further, Alkan et al. investigated whether the use of a suction drain to evacuate an intra-articular hematoma prevents the post-operative occurrence of a knee effusion in patients who have undergone partial fat pad removal or synovectomy during arthroscopic meniscectomy [7]. They found that the use of a suction drain on the first post-operative day does not influence the formation of knee effusion.

In the current study, one factor that was found to affect the amount of drainage is the operating time. Procedures that take over 60 minutes to cause more bleeding. The procedure took longer in group I because of the time taken to place an intra-articular drain and its verification on arthroscopy. Post-operative pain was comparatively better tolerated in group I patients on day one, but from day two onward there was no difference among the groups. Both groups showed a significant decrease in pain over time (days one to seven). All patients followed a post-operative regimen of tramadol (100 mg) with acetaminophen (325 mg) - one tablet every eight hours for two to three days and SOS for seven to 10 days. Injection diclofenac 50 mg intramuscular was needed early on post-operative days zero and one), with the goals of pain relief and the ability to participate in physical therapy. We did not track the volume or frequency of drugs used after discharge for pain management as a potential confounding variable. Dhawan et al. also measured pain on a visual analog scale and it was found to be statistically different between the treatment and control groups on days one, three, and five, with the control group (no drain) reporting significantly less pain on these days [3]. On day seven, the pain trend was noted to reverse. Their result differed from ours in early post-op days and they justified that the decreased amount of pain in the group without a drain may be related to the pain from the portal site from where the drain was taken out as a foreign body response, which was an interesting finding.

Further, Clifton et al. conducted a systemic review of randomized control trials of previous studies on 349 patients [6]. They found that the difference in pain scores varied among studies, with a few reporting increase in pain in the drain group and others reporting decreases in post-operative pain, while most found no significant difference.

The range of motion was measured in terms of loss of flexion and extension. Flexion of the knee was initiated only after removal of the drain on the second day, and isometric quadriceps and hamstring exercises with ankle pumps were initiated immediately after recovery from anesthesia. However, extension loss was measured from day one onward. Mahajan and Kataria divided 44 patients into intra-articular versus no drain groups and found that the loss of flexion was comparable in both the groups during the entire rehabilitation period, which was similar to our study [9]. At the six-month follow-up, we found no flexion loss and patients were able to squat normally. Karhan et al. conducted a randomized control trial on 34 patients that involved placing a drain at the donor site versus no drain at the donor site and compared the functional outcome [10]. They found that there was no difference in the extension in the drain and non-drain groups.

No major complications were noted in our study during either the intra-operative or post-operative periods, including infection at the surgical site, graft avulsion, or tourniquet palsy. Four of our patients (9.09%) developed cutaneous anesthesia over the distribution of the saphenous nerve immediately in the post-operative period, which was attributed to the saphenous nerve location near the graft harvesting incision. This happened despite an oblique incision in all our patients, which is expected to reduce the incidence of this type of anesthesia.

## Conclusions

From our study, we can conclude that putting an intra-articular drain after ACL reconstruction is beneficial only in the early post-operative period, while in the long-term it offers no advantage in terms of functional outcome. However, we also found that the use of the drain at the donor site, which was only used as a blinding method, may also have advantages in the reduction of pain and supra-patellar girth. Additional studies are required to evaluate the effect of a drain at the donor site. 

Another significant observation is that none of our patients developed a complication related to the use of the drain, despite the drain having been used in both the groups; therefore, concern regarding drain-related complications may be overstated. However, this aspect could also be investigated with a larger sample in future research.
